# Prediction of protein interactions between pine and pine wood nematode using deep learning and multi-dimensional feature fusion

**DOI:** 10.3389/fpls.2024.1489116

**Published:** 2024-12-02

**Authors:** Liuyan Wang, Rongguang Li, Xuemei Guan, Shanchun Yan

**Affiliations:** ^1^ College of Computer and Control Engineering, Northeast Forestry University, Harbin, Heilongjiang, China; ^2^ Key Laboratory of Sustainable Forest Ecosystem Management, School of Forestry, Northeast Forestry University, Harbin, Heilongjiang, China

**Keywords:** protein-protein interaction, pine wilt disease, deep learning, multi-dimensional feature, pine wood nematode (*Bursaphelenchus xylophilus*)

## Abstract

Pine Wilt Disease (PWD) is a devastating forest disease that has a serious impact on ecological balance ecological. Since the identification of plant-pathogen protein interactions (PPIs) is a critical step in understanding the pathogenic system of the pine wilt disease, this study proposes a Multi-feature Fusion Graph Attention Convolution (MFGAC-PPI) for predicting plant-pathogen PPIs based on deep learning. Compared with methods based on single-feature information, MFGAC-PPI obtains more 3D characterization information by utilizing AlphaFold and combining protein sequence features to extract multi-dimensional features via Transform with improved GCN. The performance of MFGAC-PPI was compared with the current representative methods of sequence-based, structure-based and hybrid characterization, demonstrating its superiority across all metrics. The experiments showed that learning multi-dimensional feature information effectively improved the ability of MFGAC-PPI in plant and pathogen PPI prediction tasks. Meanwhile, a pine wilt disease PPI network consisting of 2,688 interacting protein pairs was constructed based on MFGAC-PPI, which made it possible to systematically discover new disease resistance genes in pine trees and promoted the understanding of plant-pathogen interactions.

## 1 Introduction

Pine Wilt Disease (PWD) is a devastating and prevalent forest disease caused by the pine wood nematode (*Bursaphelenchus xylophilus*), which is known as the “cancer” of pine trees and the “avian flu” of pine forests. The disease can harm most pine (*Pinus*) plants, and it can destroy entire pine forests within 3 to 5 years from the initial infection, the pine forest resources, natural landscape and ecological environment caused serious damage (Xu et al., 2023). Therefore, exploring the pathogenic mechanisms of pine wilt disease and achieving effective early prevention have become top priorities in global forest protection.

Protein-protein interactions (PPI) between plants and pathogens are fundamental to understanding infection mechanisms and host response strategies. The first line of defense for plant disease resistance is to recognize pathogen-associated molecular patterns (PAMPs) through cell surface receptors (PRRs), which activates pattern-triggered immunity (PTI) (Yuan et al., 2021). At this stage, to destroy the host immune system, pathogens will secrete effector proteins that interact directly or indirectly with plant proteins, interfering with the plant’s PTI response. To counteract pathogen virulence, plants initiate a second line of defense that specifically recognizes effectors through intracellular resistance proteins (R), thereby activating effector-triggered immunity (ETI) (Naveed et al., 2020). In summary, the interactions between host resistance proteins and pathogen-effector proteins play a pivotal role in plant-pathogen molecular recognition (Cardoso et al., 2024). The interaction between the pine wood nematode and pine tree serves as a quintessential model of plant-pathogen relationships, encompassing both the processes of pathogen infection and destruction of the host, as well as host perception and defense against invasion. Therefore, investigating the protein-protein interaction networks between the pine wood nematode and its host pine trees is crucial for elucidating the pathogenic mechanisms of pine wood nematode disease. This understanding is of great significance for achieving early pest control, maintaining forest health and ecosystem balance.

Traditional methods for protein-protein interactions identification, including yeast two-hybrid screen(Y2H) (Uetz et al., 2000), affinity purification-mass spectrometry (AP-MS) (Gavin et al., 2002), and co-immunoprecipitation (Co-IP) (Bennett et al., 2010), were initially widely used in human-virus PPI studies (Zhou et al., 2023; Kim et al., 2023). These techniques laid the foundation for phytopathology network studies, resulting in a series of plant-pathogen PPI databases such as HPIDB (Ammari et al., 2016), PHI-base (Winnenburg et al., 2007), and UVPID (Jiehua et al., 2019). However, these methods are time-consuming and costly, which make the study of disease resistance mechanisms in non-model plants generally lack of holistic nature, and experimentally validated PPIs between Pinus sylvestris and Pinus sylvestris nematodes are even fewer (Meng et al., 2017; Liu et al., 2021). Consequently, there is an urgent need to develop a fast and accurate plant-pathogen PPI prediction method to elucidate the pathogenic system of pine wilt disease.

Computational prediction methods for PPIs play an increasingly important role benefiting from the rapid development of computational biology. Methods based on machine learning (ML) have been widely applied in early computational modeling studies of cross-species PPI (Tang et al., 2023). A range of ML methods, have demonstrated effectiveness in PPI prediction tasks for species like human hepatitis C virus and Arabidopsis (Wang et al., 2021; Ahmed et al., 2021; Lei et al., 2023). With the increasing availability of high-throughput sequencing data, traditional ML methods are becoming inadequate for handling vast amounts of data, and deep learning (DL) has attracted wide attention in the field of bioinformatics due to its powerful model expression ability (Zhang et al., 2024). Protein sequences serve as the primary data source for PPI prediction, and many models leverage sequence information to conduct predictive research, such as DNN-PPI (Li et al., 2018), DeepFE-PPI (Yao et al., 2019), PIPR (Chen et al., 2019), and so on. A series of results have also been achieved in plant-pathogen PPI prediction research work, for example, Zheng (Zheng et al., 2023) fused protein sequence, structural domain and gene ontology (GO) information to construct a deep learning framework based on the combination of word2vec and RCNN to predict protein-protein interactions in *Arabidopsis thaliana*, and validated its ability to cross-species prediction in different datasets. Pan (Pan et al., 2022) fuses protein sequence information with behavioral information to predict interactions between different plant proteins using DNN and obtains more than 92% accuracy in a variety of datasets. Li (Li et al., 2022) proposes a plant-pathogen prediction model by combining position-specific scoring matrices (PSSMs) and evaluates the effectiveness of the model in *Arabidopsis thaliana*, *Zea mays*, and *Oryza sativa* datasets.

Recently, the general interest of researchers in predicting PPI based on structural information has been driven by the rapid growth of three-dimensional (3D) structural data of proteins, especially by the vacated introduction of AIphaFold (Jumper et al., 2021; Varadi et al., 2022), a series of structural and graphical neural network-based prediction models have emerged, such as DensePPI (Halsana et al., 2023), GraphPPIS (Yuan et al., 2022), TAGPPI (Song et al., 2022), and Struct2Graph (Baranwal et al., 2022). Zheng (Zheng et al., 2021) proposed a computational framework based on structural and homology modeling, which not only predicted the PPI networks of rice and the rice blast fungus, generating an interactions network with 2,018 protein pairs, but also analyzed the network to make systematic discovery of plant disease resistance genes possible. Improvement of PPI prediction in combination with AIphaFold may be a solution to this problem by making predictions based on primary structure, which in turn results in more protein structural information that can be used on a genome-wide scale (Homma et al., 2023; Bryant et al., 2022).

In summary, the structural information of proteins is more conserved relative to sequences during evolution and can be obtained with higher accuracy, but the sequence evolution information of proteins is crucial for predicting functionally relevant interactions. Models relying solely on structural information might overlook such vital biological data, limiting their applicability and generalization capability (Kang et al., 2023; Vajdi et al., 2020). Therefore, an approach that fuses sequence and structural features by integrating a deep learning framework can reveal the details of protein interactions, which can be beneficial for generalization to species-wide prediction efforts and elucidation of genome-to-phenomenon (Sledzieski et al., 2021).

In this study, Graph Convolutional Networks (GCN) and attention mechanisms focused on critical interaction nodes, combined with AlphaFold, were used to predict the PPIs between pine wood nematodes and their host pine trees. Compared to the sole use of amino acid sequences or protein structure data, the proposed method took a multi-level feature fusion approach to acquire more comprehensive protein representation information, thereby reducing evolutionary differences in cross-species PPIs and improving predictive accuracy. Moreover, by converting structural data into graph data, it was represented in the form of graph theory to identify new interaction residues in the plant-pathogen system in an unsupervised manner. In addition, PPIs for pine and pine wood nematode proteins were constructed using a multidimensional feature fusion method, providing valuable insights for systematically understanding plantpathogen interactions and the pathogenic mechanisms of Pine wilt disease.

## 2 Materials and methods

### 2.1 Construction of datasets

High-quality training data is crucial for deep learning models, but the availability of known PPI data between plants and pathogens is very limited. Therefore, training models on known protein interaction datasets to predict PPIs in new host-pathogen systems becomes particularly important. In this study, to improve the accuracy of the model while preserving the biological significance to the greatest extent, protein-related data of pine nematode and host pine as well as PPI data verified by biological experiments, were selected to build a dataset suitable for training a pine nematode-pine PPIs prediction model.

First, the raw protein sequence data for both the pine wood nematode and pine tree, which served as the basis for subsequent construction of protein 3D structures and extraction of sequence features, was obtained. The protein sequence data for the pine wood nematode was gained from the NBIC database (Taxonomy ID: 6326), totaling 53,412 sequences. The protein sequence data for the pine tree was obtained from UNIPROT(https://www.uniprot.org), totaling 200,806 sequences. The genome annotation project database for pine trees was TreeGenes (https://www.treegenesdb.org). To minimize potential errors from raw protein sequence data, sequences containing short protein sequences (e.g., lengths less than 50 bp) and homologous sequences were removed.

The experimentally validated protein-protein interaction data between the pine wood nematode and its host pine tree was gained from the PHI-base (https://www.phi-base.org) database.

PPI data for both the pine wood nematode and pine tree were queried from the STRING database. Specifically, there were 6,231 interaction pairs for the pine wood nematode and 2,009 interaction pairs for the pine tree. All interaction data selected here were physical interactions, excluding weak and transient interactions. The interaction data between the two were analyzed and compared, and the overlapping data was regarded as interspecific interaction. Finally, combining data obtained from PHI-base, 8,259 protein-protein interaction pairs were constructed using 4,792 proteins, including 5,258 positive reference datasets. It is worth noting that protein interactions can take various forms, only direct physical interactions were considered as positive data. Non-interacting proteins cannot be directly obtained from PPI databases, so proteins with no interactions in the PPI network were marked as negative samples not recorded in the PPI dataset.

AlphaFold can obtain the representation information of 3D structure through protein sequences. Therefore, AlphaFold DB would be used to view the structural information of protein interactions generated based on the above rules, and structure prediction would be made for protein sequences lacking structural information to generate PDB files to establish a protein structure database for PPI prediction of pine wood nematode disease.

According to the structural graph of protein network interactions, an interaction between two proteins was marked as 1, otherwise as 0. There were 8,259 positive and negative samples in total, which were divided into training, testing sets in an 8:2. The validation set was used to determine the optimal parameters for the model, and the trained model with the determined optimal parameters was used for training. To improve model performance, different proportions of positive and negative samples were set, and 1/2 of the positive (negative) samples from the training set were mixed with the negative (positive) samples from the training set to enhance the model’s generalization capability under imbalanced sample conditions.

### 2.2 Network overall framework

**Figure 1:**
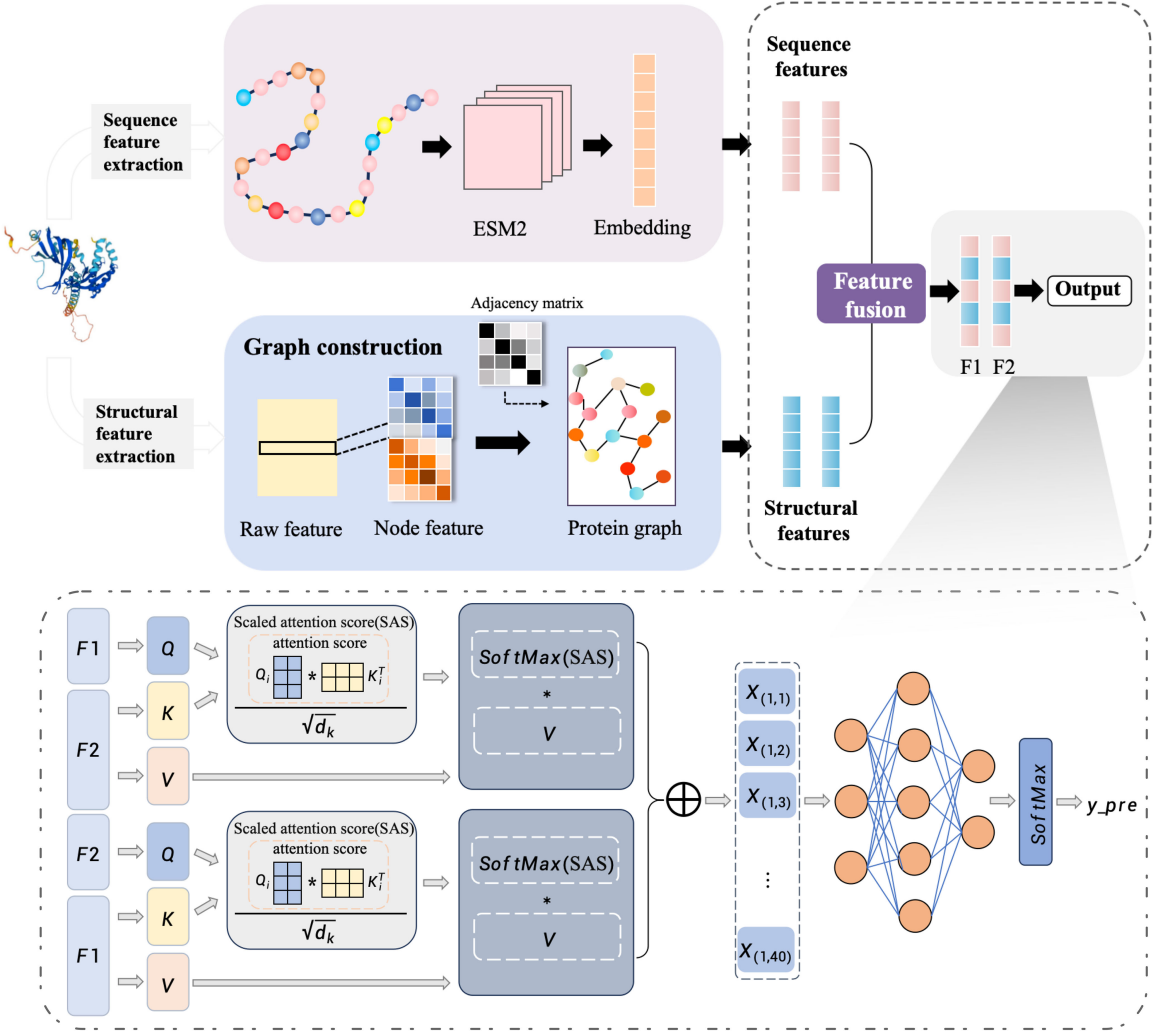
Overall structure of the model.

An end-to-end deep learning framework MFGA-PPI was introduced for identifying plant-pathogen PPIs. Here, we take the sequence and structure information of the two proteins as input and define it as a binary classification problem, with the final output being a set of 0 or 1 predictions as to whether they interact or not. The overall architecture of the MFGAC-PPI model is shown in **Figure 1**. This model architecture consisted of three parts: the feature extraction module, the feature aggregation module, and the prediction module. In the first part, to better represent protein structures, both tertiary and primary structure information and design feature extraction modules were used for each. The second part used a linear interpolation method to effectively combine the two feature vectors, achieving a multidimensional protein representation. The third part inputed the resulting protein pairs into an attention network, calculated attention scores, and used them for PPI prediction.

#### 2.2.1 Construction of graph data

After encoding the 3D structural features, the spatial information of protein pairs needed to be converted into corresponding protein graphs, and inputed into the graph neural network for processing.

The protein feature coding process was divided into two parts: extraction of tertiary structure features and extraction of primary structure features.

As shown in **Figure 1**, the protein feature encoding process was divided into two parts, i.e., extracting tertiary structure features and extracting primary structure features. Firstly, the main chain of the protein was selected for feature extraction. Due to the different atoms composing the 20 amino acids, each atom had distinct characteristics in different amino acids.

According to the atoms that made up the amino acid residues, four atomic features, namely van der Waals radius, electronic charge, B-factor and atomic mass, were extracted from the PDB file of the tertiary structure, and are used to represent ${\left\{F_{i,j}\right\}}_{i=1,\text{...},4;j=1,\text{...},N}$ , where $\left\{F_{i,j}\right\}$ represents the *i*th feature of the *j*th atom in the residue. Then, the average of each atomic feature within the residue is calculated and represented by ${\left\{f_{i}\right\}}_{i=\;1,\ldots ,4}$ , where *i* represents the *i*th atomic feature of the residue, and $f_{i}$ indicates the average feature value of all atoms in the residue. Finally, a four-dimensional atomic feature for each residue was obtained. The calculation formula is as follows:


(1) $$f_{i}=\frac{1}{N}\sum_{j=1}^{N}F_{i,j}$$

After the atomic features were obtained, the geometric structure of the protein was analyzed to measure the distances between residues and assess the possibility of interactions.

There are various methods to construct geometric shapes based on atomic spatial coordinates. Borgwardt (Borgwardt et al., 2005) described spatial contacts between atoms through van der Waals forces or hydrogen bonds. Based on the idea of graph theory, Cha (Cha et al., 2022)took the atomic positions after averaging as the spatial position coordinates of amino acids and regarded them as the vertices of the graph.

Given that graph theory-based methods can reveal the topological structure of protein networks, the concept of graph theory was firstly adopted by aggregating amino acids based on group numbers and calculating the average distance between atoms within the group to obtain the coordinates of each residue. Subsequently, using the residue coordinates, the pairwise distances between residues were calculated using the Euclidean distance. Generally, if the distance between two amino acids in space was less than 8 Å, they were considered to be in contact. Therefore, a spatial proximity relationship was determined if the distance between residues was less than 8.0 Å (Mou et al., 2023). The protein graph of length *l* is a square matrix $C=\;\left\{c_{a},b\right\}$ of order *l*, where


(2) $$C_{a,b}=\{\begin{array}{l} \begin{array}{ll} 1 & if\;\alpha_{a,b}<8.0Å\\ 0 & otherwise \end{array} \end{array}$$

Finally, we construct an *n* × *n* residue matrix was constructed and the amino acid residues to unique integer identifiers were mapped, being added to the atomic features to generate the node feature matrix $X_{v}\in {\mathbb{R}}^{n\times 4}$ , where *n* is the number of residues and 4 represents the four-dimensional node vector extracted. The constructed protein graph object can be denoted as G= (V,E,A) , where *V* is the set of vertices, *E* is the set of edges between them, vertices represent amino acid residues, edges represent spatial proximity relationships between residues, and *A* is the adjacency matrix, $A\in {\mathbb{R}}^{l\times l}$ .

Protein sequence features were extracted based on the main chain, which typically consists of three atoms: nitrogen (N), alpha-carbon (C*α*), and carbonyl carbon (C). The alpha-carbon (C*α*) atom is commonly used to mark the position of amino acids (Lin et al., 2022). The ESM-2 model was employed to extract features from protein sequences. ESM2 is a language model based on the Transformer architecture, which maps protein sequences to representations in a high-dimensional space (Liu and Shen, 2023). First, the coordinates of the C*α* in the protein structure information were used as reference points for amino acids and traversed each main chain and residue to obtain the amino acid sequence. Then, we use the ESM2 model was used to encode the protein sequence, mapping each protein sequence to a 1280-dimensional vector and obtaining embeddings at the protein level. Finally, the sequence embeddings were added to the graph embeddings computed by the GCN block, which were then normalized to the final output embeddings to obtain the sequence feature vector $F_{g}\in {\mathbb{R}}^{1\times 20}$ .

#### 2.2.2 Improved graph convolutional network processes protein graph

GCN is a convolutional neural network for processing graph data, where nodes represent residues and edges represent relationships between residues. Here the features of different nodes in the protein graph data are aggregated using the improved GCN module, which continuously learns and updates the node features.

Node features first enter a 1D convolution layer to extract local features, followed by processing through a bidirectional gated recurrent unit (GRU) to capture global dependencies of the residue node features and output a feature sequence. Finally, an average pooling layer compressed the GRU output to generate fixed-size feature representations. For the stability of convolution operations, the adjacency matrix *A* is normalized. The feature was propagated to the neighbor node through the adjacency matrix *A*1 and *A*2, which were the adjacency matrices of two proteins respectively, and the updated feature of this node containing the neighbor node was saved. The main calculation formula of GCN module is as follows:


(3) $$H^{\left(l+1\right)}=\sigma \;\left({\tilde{D}}^{-\frac{1}{2}}\;\tilde{A}{\tilde{D}}^{-\frac{1}{2}}\right)H^{\left(l\right)}W^{\left(l\right)}$$

where $\tilde{A}=A+I$ , *A* is the adjacency matrix and *I* is the identity matrix. Similarly, $\tilde{D}=D+I$ , where *D* is the degree matrix of *A*, and *I* is the identity matrix. $H^{\left(l\right)}$ represents the updated features of the *l*-th layer residue nodes. When *l* = 0, it indicates that the node has not been updated, thus $H^{\left(0\right)}=X_{v}$ , where $X_{v}$ represents the initial residue node features. $W^{\left(l\right)}$ is the trainable weight matrix of the *l*-th layer, and σ() is the nonlinear activation function. The feature of the last updated residue node was inputted into $F_{g}$ , and a total of 3 residue node updates were carried out in this experiment.

Therefore, for a pair of proteins A and B, we can extract richer structural feature vectors can be extracted as $F_{1g}$ and $F_{2g}$ , $F_{1g}\in {\mathbb{R}}^{1\times 20}$ , $F_{2g}\in {\mathbb{R}}^{1\times 20}$ . Finally, the resulting structural feature vectors were combined with the ESM output $F_{1s}$ and $F_{2s}$ to obtain F1 and F2 , where $F1=F_{1g}+\text{λ}F_{1s}$ , and $F2=F_{2g}+\text{λ}F_{2s}$ , with λ being an adjustable parameter.

#### 2.2.3 Attention network for PPI prediction

The scaled dot-product attention mechanism was employed to compute the attention between F1 and F2. A scaling factor was introduced before the dot-product calculation to balance the magnitude of the results, evaluating which residues play critical roles in the interaction.

When calculating the attention of *F*1 on *F*2, the scaled dot-product attention primarily accepted three parameters: Query (*F*1), Key (*F*2), and Value (*F*2). First, the attention score(AS) was computed by taking the dot product of the query and key matrices. Secondly, to control the range of attention scores and prevent gradient explosion or vanishing, the key vector dimension $d_{k}$ was used to scale it and the scaled attention score (SAS) was obtained. Finally, the attention weight matrix was multiplied by the value matrix to compute the weighted sum. The formula of the attention network is as follows:


(4) $$SA=F1\times F2^{T}$$

(5) $$SAS=\frac{F1\times F2^{T}}{\sqrt{d_{k}}}$$

(6) $$S_{1}=softmax(\frac{F1\times F2^{T}}{\sqrt{d_{k}}}),\;S_{2}=softmax(\frac{F2\times F1^{T}}{\sqrt{d_{k}}})$$

The final attention scores $S_{1}$ and $S_{2}$ were concatenated to obtain $s=\;\left[S_{1},S_{2}\right]$ , which served as the output of the attention mechanism.

#### 2.2.4 FNN layer

A fully connected layer was used to predict the model output. The vector s obtained from the attention layer was used as the input to the fully connected layer, which produced the vector $z=\;\left[Z_{1},Z_{2}\right]$ . Finally, a softmax activation function was applied to yield the binary classification result.


(7) $$y\underline{\;}pre=softmax\left(z\right)=\left[\frac{e^{z_{1}}}{e^{z_{1}}+e^{z_{2}}},\frac{e^{z_{1}}}{e^{z_{1}}+e^{z_{2}}}\right]$$

### 2.3 Model training and hyperparameter setting

In this study, Intel(R) Xeon(R) Gold 6354 CPU @ 3.00GHz×2, NVIDIA GeForce RTX 3090×4 graphics processor, 256.0GB memory, 19TB MR9364-8i storage were used, and operating system was Ubuntu 18.04.4 LTS.

In the process of model training, 5-fold cross-validation was adopted. For each fold training set, 1000 samples were randomly selected for training. For each selected sample, the characteristics and labels of the sample were obtained. In this experiment, the Adam optimizer was used to optimize the model. The initial value of the learning rate was 1 × 10^−3^, and the learning rate attenuated to half of the original value after every 10 rounds of training to prevent the model from jumping out of the optimal solution. In this model, three layers of GCN neural networks are used, and the embedding dimension of GCN in each layer was 20, which reduces the computational overhead on the premise of ensuring sufficient feature information capture. The feature dimension of the protein sequence captured by ESM was 1280 dimensions. Binary cross-entropy is used as the loss function of the model in this paper, and the formula is as follows:


(8) Loss=(−y log(p)+(1−y) log(1−p))

where, *y* is the true label and *p* is the probability that the model predicts 1. *y* log(*p*) represents the loss when the true label is 1 and (1 − *y*) log(1 − *p*) represents the loss when the true label is 0.

### 2.4 Evaluation metrics

In the experiment, eight common indicators were used to evaluate the model: Precision, Recall, Accuracy, Specificity, F1-score, Matthews Correlation Coefficient (MCC), Area Under the P-R Curve(AUPRC) and Area Under the ROC Curve (AUROC). Their relevant definitions are as follows:


(9) $$\begin{array}{c} Precision=\frac{TP}{TP+FP},Recall=\frac{TP}{TP+FN} \end{array}$$

(10) $$\begin{array}{c} Accuracy=\frac{TP+TN}{TP+FN+FP+TN} \end{array}$$

(11) $$\begin{array}{c} Specificity=\frac{TN}{TN+FP},F1=\frac{2\times Precision\times Recall}{Precision+Recall} \end{array}$$

(12) $$\begin{array}{c} MCC=\frac{TP\times TN-FP\times FN}{\sqrt{\left(TP+FP\right)\left(TP+FN\right)\left(TN+FP\right)\left(TN+FN\right)}} \end{array}$$

## 3 Results

### 3.1 Ablation experiment

The basic framework of the MFGAC-PPI model mainly consisted of four submodules: ESM, improved GCN, Scaled dot-product attention, and FNN. According to the aforementioned hyperparameter settings, each submodule was treated as a different variable and the “control variable method” was used to investigate the influence and contribution of each submodule to the proposed model. Therefore, 5-fold cross-validation was used to conduct ablation studies on these four structures, six indicators were used for evaluation, and the maximum value of the results of each ablation was selected, as shown in **Table 1**. It can be seen that, when any of the sub-modules was ablated, the overall prediction performance of the model was degraded, indicating that each submodule played a role, and the structural design was reasonable without structural redundancy. Similarly, it was found that, the model performance decreases the most among the evaluation metrics when the improved GCN module was ablated, especially specificity and MCC, which dropped by 1.73% and 2.19%, respectively. In contrast, when the FNN module was ablated, the model performance changed the least across all metrics, with precision only dropping by 0.01%, and the largest change being in MCC, which decreased by 1.39%.

**Table 1:** The performance results for different modules.

Module ablation	Accuracy	Precision	Recall	Specificity	F1	MCC
Attention	0.9779	0.9915	0.9796	0.9770	0.9816	0.9400
ESM	0.9751	0.9887	0.9756	0.9770	0.9802	0.9431
GCN	0.9723	0.9836	0.9741	0.9655	0.9761	0.9340
FNN	0.9779	0.9915	0.9798	0.9809	0.9638	0.9420
All	**0.9812**	**0.9916**	**0.9893**	**0.9828**	**0.9904**	**0.9559**

*Best performance is shown in bold.

From the results, it was evident that the improved GCN module contributed the most to the overall model and significantly affected its prediction performance. In contrast, the FNN had the least impact on the predictive ability of the model.

**Figure 2:**
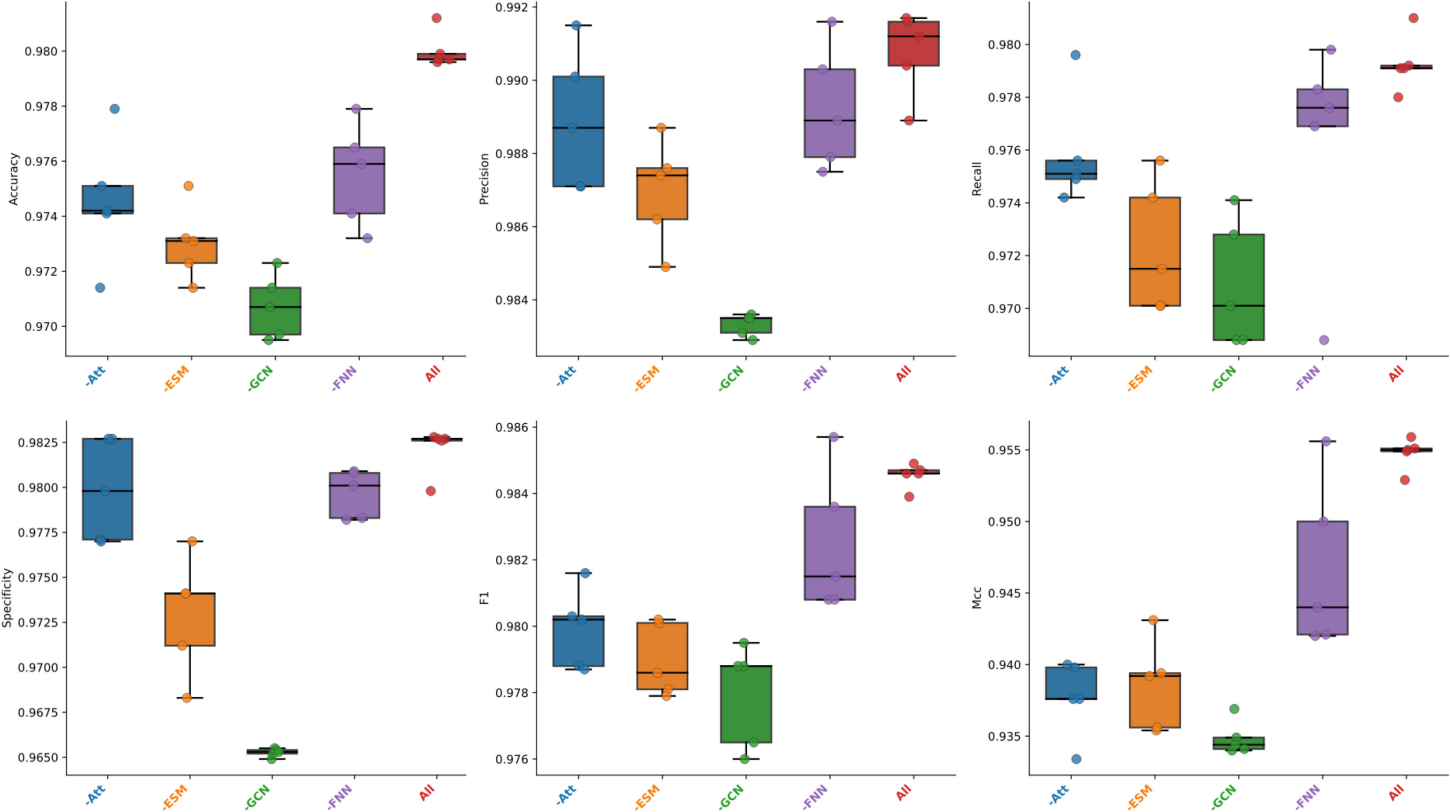
The 5-fold cross-validation of performance results.

To assess the importance of each sub-module more intuitively and comprehensively, the results of the five-fold cross-validation in each fold of the six evaluation metrics are presented here using box plots, as shown in **Figure 2**. It can be visually observed from the figure that when the improved GCN was ablated, the model showed the lowest average values and the lowest outliers across all metrics. The model without FNN showed the most stable performance in the five-fold cross-validation, with the highest average values across all metrics. However, in precision and specificity, it had nearly the same values as the model without the attention module, but the model without attention had lower dispersion points. In summary, each sub-module had an important contribution and influenced the model’s effectiveness to some extent. Among them, the improved GCN contributed the most to the model’s overall performance, followed by the ESM module, both of which reflected the importance of feature fusion to some extent. Then there was attention and FNN, with FNN having the smallest impact on the overall model.

The model constructed in this study differed from the GAT module by introducing a Scaled dot-product attention to capture subtle differences within proteins and their interaction interfaced while retaining more interpretable biological characteristics, which helped to identify amino acid residues with important functions, and had better robustness when dealing with proteins of different lengths. To validate that the introduction of the scaling factor improved the predictive ability of the model, as well as to explore the impact of different attention mechanisms on the model, three new models were added to the original structure: self-attention replacing scaled dot-product attention, mutual-attention replacing scaled dot-product attention, and multi-head-attention replacing scaled dot-product attention. Keeping other sub-modules and hyperparameters unchanged, these models were subjected to ablation experiments using 5-fold cross-validation, and evaluated using three comprehensive metrics: specificity, F1, and MCC for evaluation. **Table 2** shows the maximum values in the cross-validation.

**Table 2:** The performance results of different attention modules.

Attention module	Specificity	F1	MCC
Mutual-Attention	0.9743	0.9795	0.9370
Self-Attention	0.9798	0.9842	0.9545
Multihead-Attention	0.9655	0.9808	0.9418
All	**0.9828**	**0.9847**	**0.9559**

*Best performance is shown in bold.

**Figure 3:**
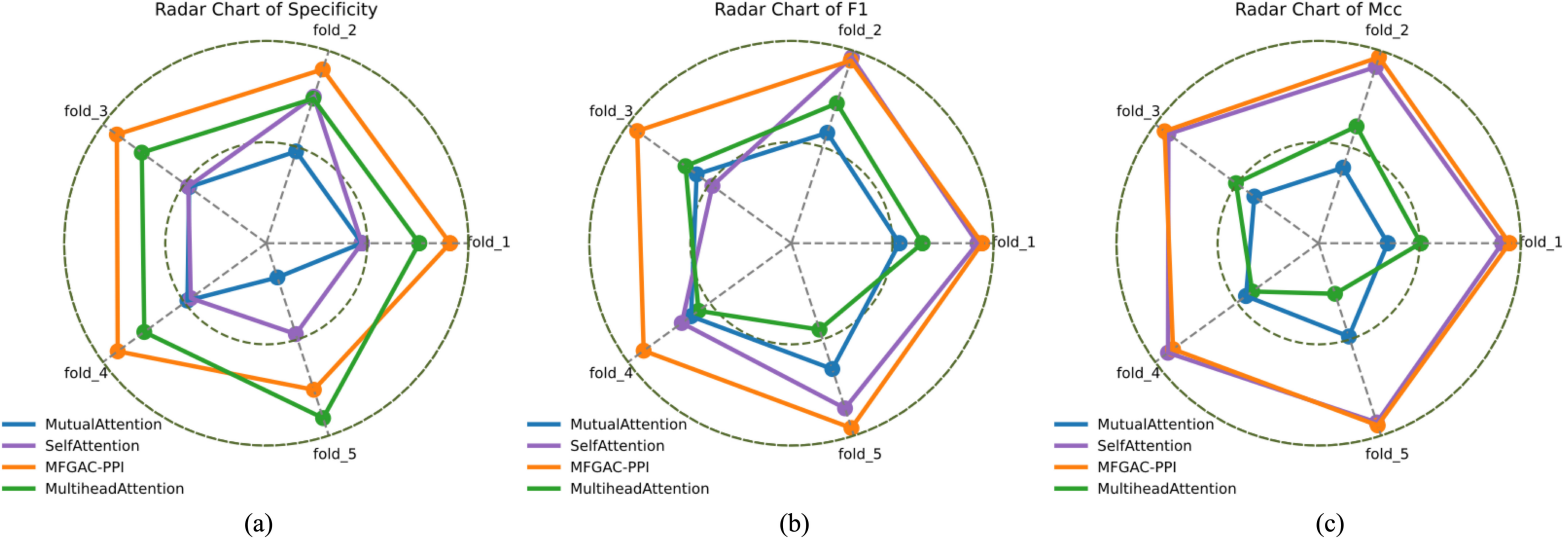
The 5-fold cross-validation radar map. **(A)** specificity 5-fold cross-validation radar map. **(B)** F1 5-fold cross-validation radar map. **(C)** MCC 5-fold cross-validation radar map.

To clearly illustrate the variation of each fold value in cross-validation, radar charts of Specificity, F1, and MCC on the validation dataset were plotted as shown in **Figure 3**. It can be seen that the model using mutual-attention had the smallest area in the metrics, while the MFGAC-PPI model using the scaled dot-product attention mechanism had the largest area in the three comprehensive metrics, indicating a significant reduction in false positive (FP) samples and an increase in true positive (TP) samples. Moreover, it was apparent from **Figures 3A, C** that the Specificity indicator’s area for the multi-head-attention model was much larger than that for the self-attention model, but the area in MCC was the opposite.

This may be due to the different focus of the two attention mechanisms when processing input data. Multi-head-attention captured different feature subspaces through multiple heads, performing better on negative sample features, while self-attention uses a single attention head to compute attention globally, focusing on optimizing overall performance, thus performing better in F1 and MCC metrics.

Combining **Table 2** and **Figure 3**, it was found that the applicability of the attention modules, from highest to lowest, was scaled dot-product attention > self-attention > multihead-attention > mutual-attention.

Additionally, different λ values were tested to evaluate the contribution of sequence and structural features to the model during feature fusion. During model training, the λ value for feature fusion in the constructed MFGAC-PPI model was adjusted while keeping other parameters constant, and the results are shown in **Table 3**. It can be seen that when the λ values were set to 0.5 and 0.7, the performance of various evaluation metrics was the optimal, especially when λ=0.7, F1 and MCC reached the best.

**Table 3:** The performance results for different λ values.

λ values	Accuracy	Specificity	F1	MCC
0	0.9735	0.9565	0.9789	0.9439
0.1	0.9831	0.9743	0.9797	0.9454
0.3	0.9763	0.9655	0.9761	0.9522
0.5	**0.9812**	**0.9828**	0.9833	0.9528
0.7	0.9807	0.9733	**0.9904**	**0.9559**
0.9	0.9708	0.9742	0.9818	0.9437
1	0.9704	0.9731	0.9833	0.9419

*Best performance is shown in bold.

### 3.2 Comparison with other competitive methods

The constructed MFGAC-PPI model were compared with five classic PPI prediction models: DeepPPI (Du et al., 2017), PIPR (Chen et al., 2019), Struct2Graph (Baranwal et al., 2022), AFTGAN (Kang et al., 2023), and TAGPPI (Song et al., 2022), and the results were shown in **Table 4**. The proposed model exhibited the best performance across accuracy, precision, recall, specificity, and F1, although MCC was slightly lower than that of Struct2Graph. TAGPPI’s performance was close to the best across all metrics, particularly specificity, which was only 0.0017 lower than MFGAC-PPI. The sequence-based models PIPR and DeepPPI showed relatively low performance in MCC but maintained a relatively balanced performance across other metrics. Meanwhile, AFTGAN showed poor performance on the constructed dataset, significantly lagging behind other methods in key metrics such as F1 and recall, which were 0.1588 and 0.1555 lower than MFGAC-PPI, respectively, indicating a notable deficiency in recognizing positive samples.

**Table 4:** The performance evaluation results of different methods.

Methods	Accuracy	Precision	Recall	Specificity	F1	MCC
DeepPPI	0.9435	0.9503	0.9527	0.9702	0.9515	0.9436
PIPR	0.9609	0.9617	0.9756	0.9613	0.9609	0.9316
Struct2-Graph	0.9796	0.9830	0.9725	0.9543	**0.9777**	0.9725
AFTGAN	0.8437	0.8295	0.8338	0.9511	0.8316	0.9582
TAGPPI	0.9781	0.9710	0.9726	0.9811	0.9718	0.9525
MFGAC-PPI	**0.9812**	**0.9916**	**0.9893**	**0.9828**	0.9904	**0.9559**

*Best performance is shown in bold.

**Figure 4:**
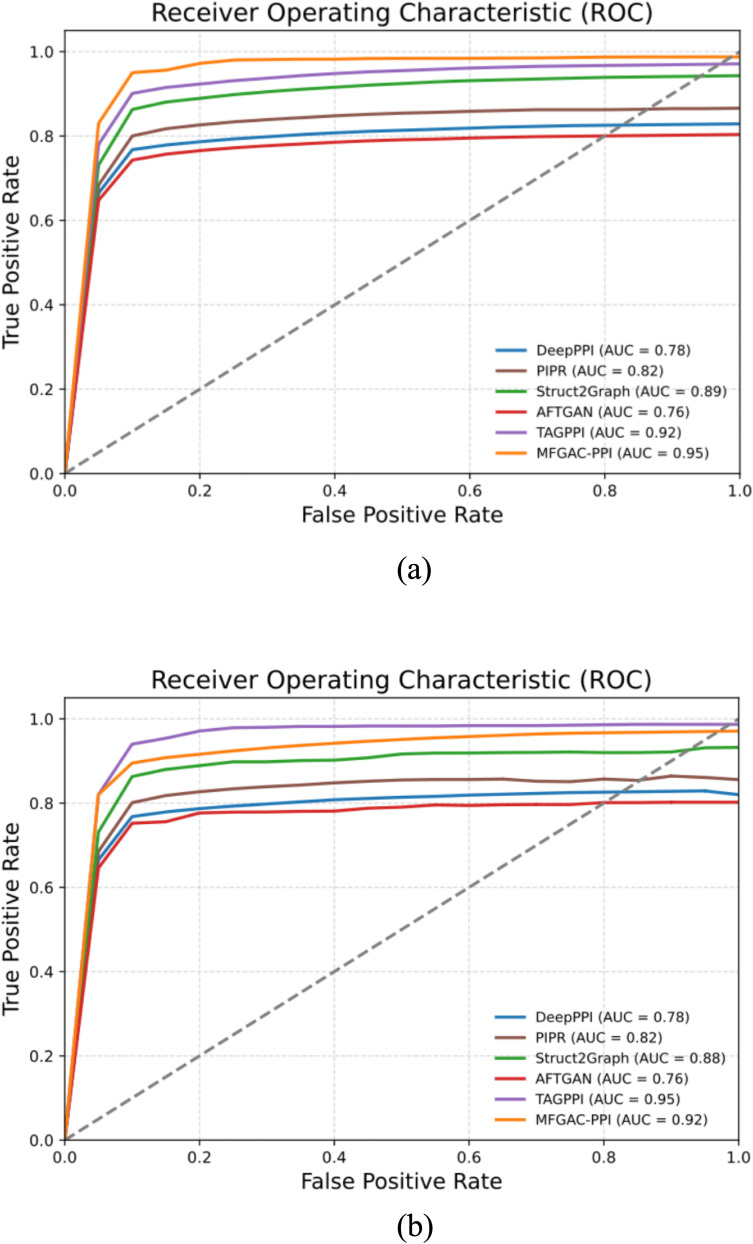
AUROC results compared with competing methods. **(A)** ROC curve verified using the dataset built in this article. **(B)** ROC curve verified using the ara data dataset.

**Figure 5:**
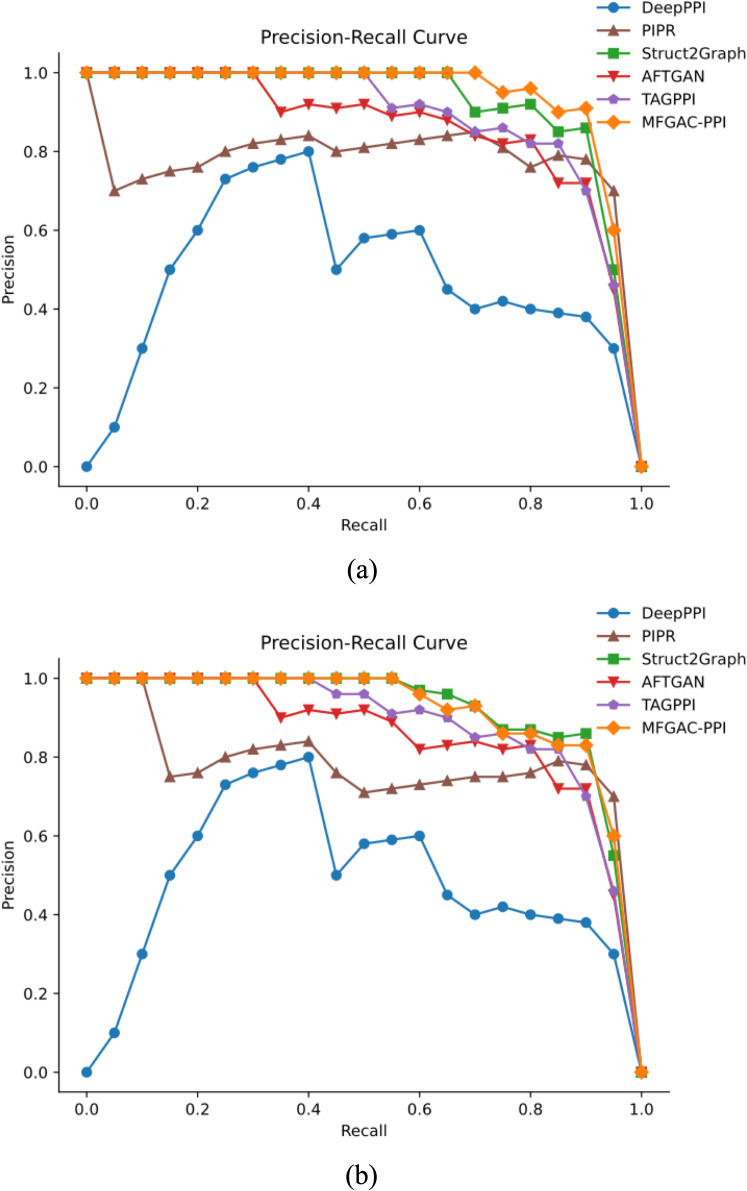
P-R results compared with competing methods. **(A)** P-R curve verified using the dataset built in this article. **(B)** P-R curve verified using the ara data dataset.

To further assess the robustness and generalization capability of the model, the proposed model was compared with five other competitive algorithms using the original dataset and an independent dataset, ara data (Zheng et al., 2023). The AUROC and P-R curves for these models on both datasets were calculated, as shown in **Figures 4**, **5**.

As shown in **Figure 4A**, in the constructed original dataset, the model’s AUC area was the largest, reaching 0.95, which was 0.03, 0.09, 0.13, 0.17, and 0.19 higher than TAGPPI, Struct2Graph, PIPR, DeepPPI, and AFTGAN, respectively. Additionally, the analysis of the P-R curve in **Figure 5A** indicated that MFGAC-PPI and Struct2Graph performed the best. The results were consistent with the performance of various metrics in **Table 4**, where the top three models in overall performance on the constructed dataset were MFGAC-PPI, TAGPPI, and Struct2Graph, respectively. As shown in **Figure 4B**, when training on the independent dataset, the AUC of MFGAC-PPI dropped by 0.03, whereas TAGPPI increased by 0.03, making TAGPPI to be the best performance in AUROC. The AUROC curves of the other models remained relatively stable, similar to the results on the original dataset, although Struct2Graph shows a slight decline. In addition, the analysis of **Figure 5B** showed that the performance of MFGAC-PPI on the P-R curve in the ara data dataset declined slightly, while Struct2Graph exhibited the best performance.

In general, MFGAC-PPI demonstrated the best performance in predictive ability and comprehensive metrics, but its performance declined when tested in different data sets, indicating that its generalization ability had room for improvement.

### 3.3 Verification of unbalanced data

In actual biological systems, most proteins interact with specific proteins rather than random combinations. Therefore, only a small fraction of protein pair combinations has true interactions, especially when PPI-related data is scarce, and the imbalance between positive and negative samples in protein interactions can reach a ratio of 1:100 or more. To verify the superiority of the proposed model in the case of unbalanced samples, the MFGAC-PPI model was trained using the constructed dataset with balanced (1:1) to unbalanced (1:3, 1:5, 1:10, 1:20, 1:30) data, using precision, recall, and MCC as evaluation metrics to illustrate the advantages of the proposed model in imbalanced datasets.

The results are shown in **Table 5**, the MFGAC-PPI model showed better performance in various indexes in unbalanced data sets. Although the performance of each index decreased with the increase of the degree of imbalance, the precision and MCC remained above 91%. At the same time, recall remained relatively stable, indicating that the model can correctly identify positive samples even under highly imbalanced conditions. Overall, the MFGA-PPI model was robust, but its overall predictive performance decreased with increasing unevenness, especially affecting precision and MCC.

**Table 5:** The performance evaluation results of unbalanced dataset.

P:N ratio	Precision	Recall	MCC
1:1	**0.9916**	**0.9893**	**0.9559**
1:3	0.9798	0.9873	0.9496
1:5	0.9655	0.9502	0.9460
1:10	0.9258	0.9455	0.9319
1:20	0.9131	0.9421	0.9201
1:30	0.9124	0.9322	0.9157

*Best performance is shown in bold.

### 3.4 Generated protein interaction network in pine wood nematode disease

The optimized plant-pathogen PPI prediction model was used to predict 20,765 sets of pine nematode and pine tree protein interaction data, and the full prediction results are shown in **Supplementary Table 1**. In particular, protein pairs with interaction scores greater than 0.5 were judged to have interactions, and a total of 2,688 pine-pine wood nematode protein-protein interaction networks were generated, which contained 46 pine wood nematode proteins and 354 pine proteins, with the results shown in **Supplementary Table 2**. Among the predicted pine-pine wood nematode PPIs results, 16 predicted PPIs were validated by previous biological experiments. Meanwhile, the comparison of different models revealed that about 19% or so of the PPIs could be derived from sequence-based prediction models, and there existed about 21% of the interaction pairs that could be predicted by structure-based methods, and there was a high degree of overlap between these two parts of the PPIs. These results indicated that a multi-dimensional feature fusion approach can effectively uncover new PPI data and was successfully applied in the study of the pine wilt disease system.

**Figure 6:**
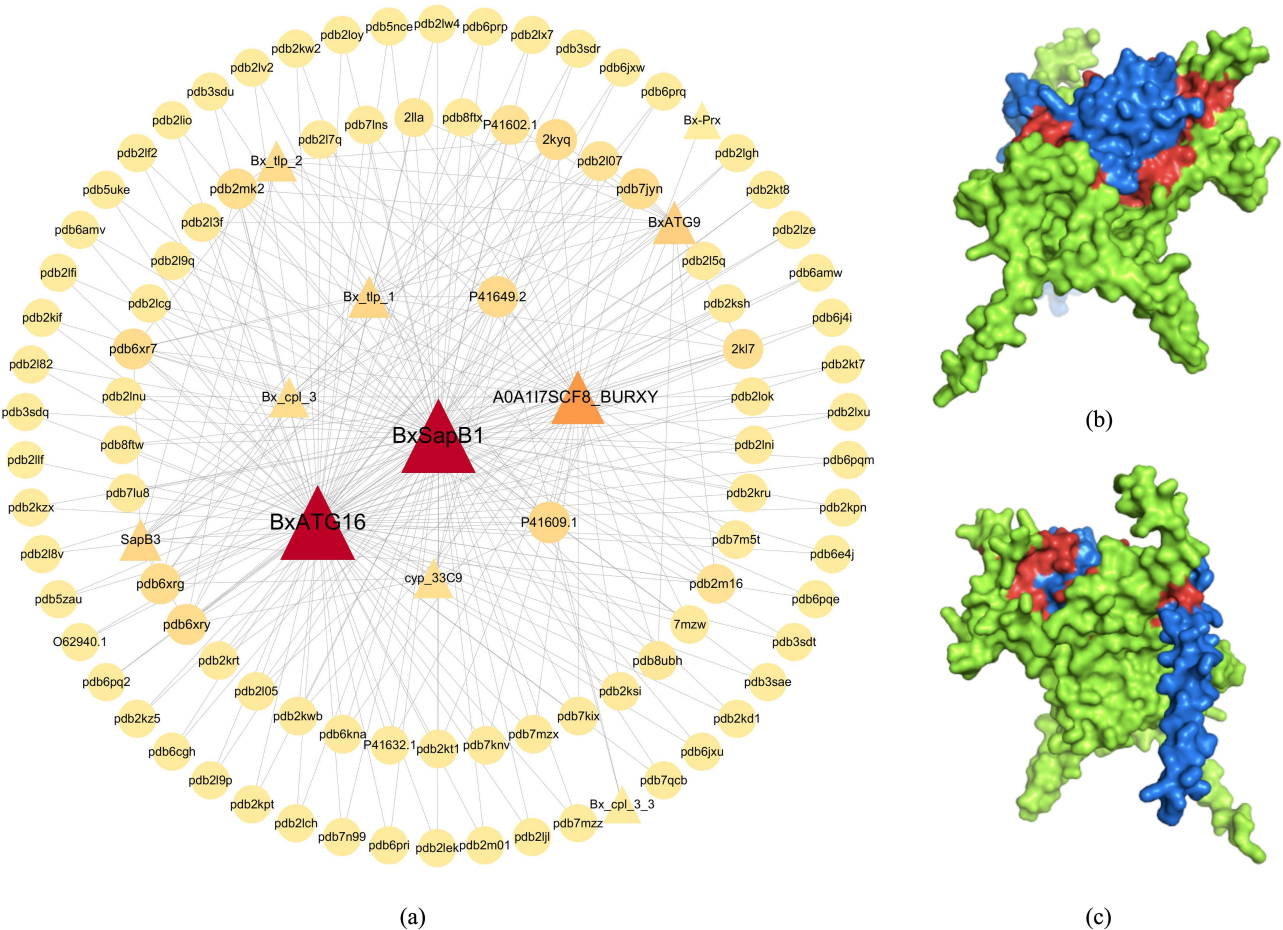
Schematic diagram of protein-protein interaction networks. **(A)** protein-protein interaction network diagram of pine wood nematode disease system. **(B)** is the schematic diagram of the interaction between effector protein SapB3 and P41649.2, and **(C)** is the schematic diagram after 180 rotation.

A topological analysis of the predicted PPI network revealed that pine-pine wood nematode PPIs exhibited scale-free properties similar to other biological networks. Notably, pine nematode proteins had more interaction links than pine proteins, with one pine nematode protein able to interact with an average of four pine proteins and at least 20 pine wood nematode proteins interact with over 10 pine proteins each. Here the nodes with higher degrees were analyzed for centrality, and the results are shown in **Supplementary Figure 1**, and a PPI network diagram was drawn as shown in **Figure 6A**. From **Supplementary Figure 1** and **Figure 6A**, it can be seen that the proteins with the most links are the effector protein *BxSap1* of pine nematode as well as the autophagy gene *BxATG16*, which played crucial roles in the virulence of the pine wood nematode. This was followed by *A0A1l7SCF8 BURXY*, *Bx tlp 1* of pine wood nematode and *P41649.2* protein of pine. This result suggested that potentially pathogen-associated proteins were more involved than resistance-associated proteins in the pine nematode system. Surface representations of the 3D structures of the interacting proteins are shown in **Figures 6B, C**, demonstrating that MFGAC-PPI can efficiently predict regions of plant-pathogen protein interaction residues, illustrated by the surface interaction between effector proteins *SapB3* (blue) and *P41649.2* (green), with the interaction region in red. By revealing 3D structural analysis and protein surface interaction regions, it helped understand molecular communication between pine trees and pathogens, explaining how pathogens evaded or suppressed the host immune response and effectively invaded host cells.

## 4 Conclusion

*Bursaphelenchus xylophilus* is one of the most devastating forest pathogens worldwide. Predicting and analyzing plant-pathogen protein interactions is a crucial step in understanding the molecular mechanisms of plant diseases. In this study, we propose MFGAC-PPI, an improved graph attention convolutional network-based deep learning method for plant-pathogen PPI prediction. It was leveraged multi-level feature fusion to provide a comprehensive research perspective and accurately predict protein-protein interactions. By utilizing AlphaFold to obtain more 3D structural information of pathogen proteins and extracting features from both amino acid sequences and structural information using the Transformer structure and GCN, the prediction accuracy was enhanced. Additionally, the scaled dot-product attention mechanism identified important interacting residues in an unsupervised manner, facilitating downstream analysis. Experimental results indicated that MFGAC-PPI achieved high accuracy on two datasets, with an AUC exceeding 92%, and performed well on imbalanced datasets. It outperformed current state-of-the-art prediction methods, making it suitable for plant-pathogen interaction prediction tasks.

Through the optimized plant-pathogen interaction prediction model, we generated a pine wood nematode disease PPIs comprising 2,688 interacting protein pairs involving 36 *Pinus* proteins and 356 *B. xylophilus* proteins. Notably, *B. xylophilus* proteins exhibited more interaction relationships and partners compared to *Pinus* proteins. This involved pathogen-related proteins in plant-pathogen interactions, potentially due to co-evolutionary arms race dynamics. The predicted PPI networks successfully identified interactions such as *BxSap1* effector with *Pinus* PR protein (*PtPR-1b*), previously validated through experimental methods (Hu et al., 2019). The results demonstrate that the MFGAC-PPI model’s successful application in the pine wilt disease system provides a comprehensive PPI network, aiding in the identification of resistance genes and advancing our understanding of plant-pathogen interaction mechanisms.

This study revealed that embedding protein sequence information into protein structural representations can extract more effective biological information, improving the accuracy of PPI prediction tasks. The performance metrics of this approach surpassed those of single-dimension protein feature learning methods. In the future, it is expected that new strategies for protein representation learning will be applied to other prediction tasks, such as protein function prediction. By combining these strategies with the protein structure prediction techniques, more high-precision structural data can be obtained, thereby expanding the model applicability and enhancing its generalization capabilities.

## Data Availability

The original contributions presented in the study are included in the article/**Supplementary Material**. Further inquiries can be directed to the corresponding author.
