# Supplementary figures and images for: Prediction of protein interactions between pine and pine wood nematode using deep learning and multi-dimensional feature fusion
Source: Front Plant Sci. 2024 Dec 2;15:1489116. doi: 10.3389/fpls.2024.1489116 (PMC11646721; doi:10.3389/fpls.2024.1489116)

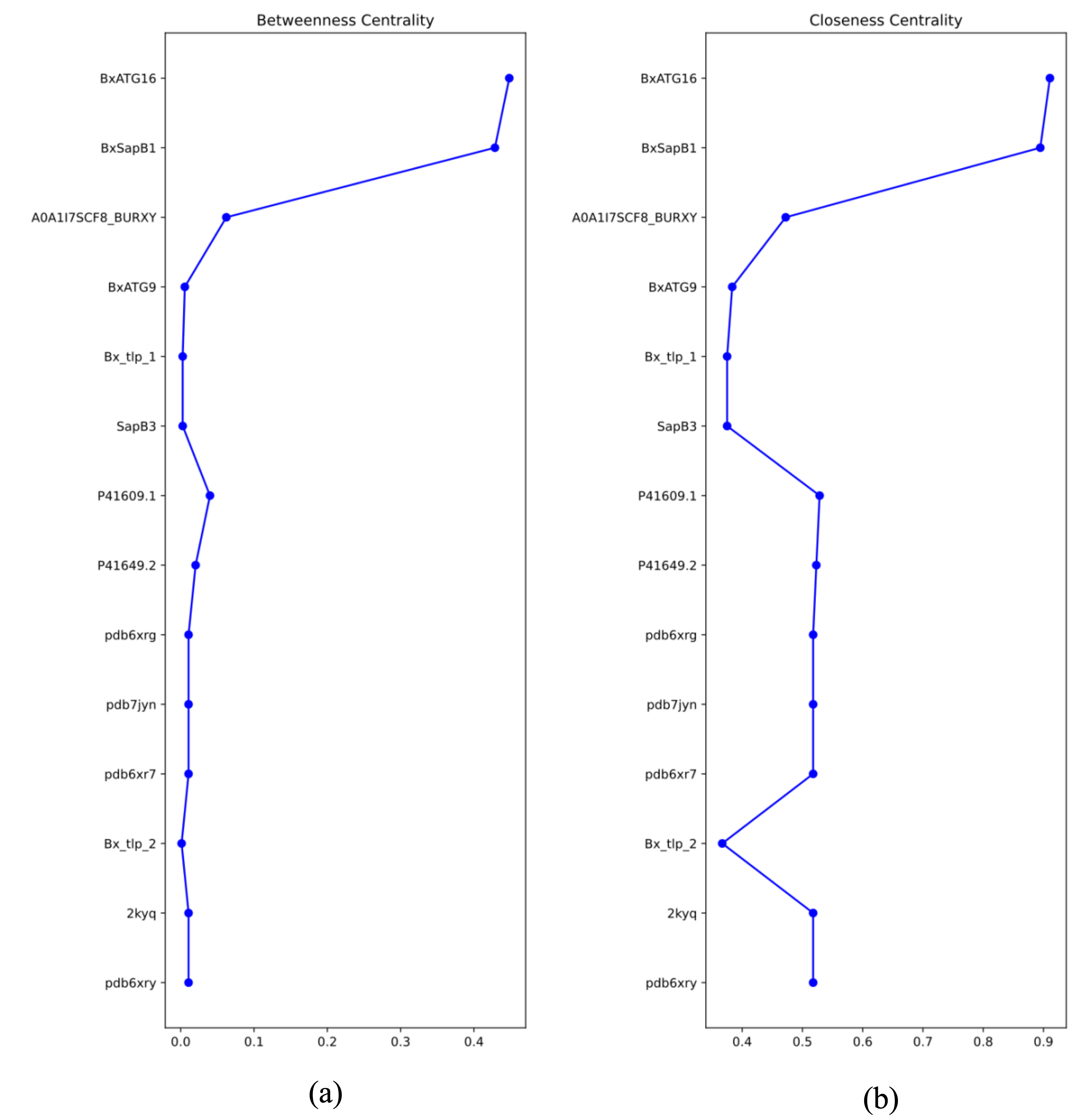

Supplement: Supplementary file 1 [file Image1.tiff]
